# Experimental and Molecular Dynamics Investigation of the Rejuvenation Effect of Bio-Oils on Aged High-Penetration Asphalt

**DOI:** 10.3390/ma18225252

**Published:** 2025-11-20

**Authors:** Hongxia Xiong, Shichao Liang, Quantao Liu, Shisong Ren, Georgios Pipintakos, Shaopeng Wu, Muyu Liu, Shi Xu

**Affiliations:** 1 School of Civil Engineering and Architecture, Wuhan University of Technology, Wuhan 430070, China; 2 State Key Laboratory of Silicate Materials for Architectures, Wuhan University of Technology, Wuhan 430070, China; 3 Sustainable Pavement and Asphalt Research (SuPAR) Group, Faculty of Applied Engineering, University of Antwerp, 2020 Antwerp, Belgium; 4 Hubei Key Laboratory of Roadway Bridge and Structure Engineering, Wuhan University of Technology, Wuhan 430070, China

**Keywords:** high-penetration asphalt, aging, rejuvenating, bio-oil, physical and rheological properties, chemical composition, molecular dynamics

## Abstract

The deterioration of high-penetration asphalt pavements due to oxidative aging presents a significant challenge in highway maintenance. This study investigates the rejuvenation effect of three bio-oils, namely palm oil, soybean oil, and sunflower oil, on aged PEN 90 asphalt through an integrated approach combining experimental characterization and molecular dynamics (MD) simulations. Laboratory evaluations, including penetration, softening point, dynamic shear rheology (DSR), and Fourier Transform Infrared (FTIR) spectroscopy, were conducted to quantify the recovery of the physical, rheological, and chemical properties of aged high-penetration asphalt. MD simulations were conducted to provide insights into diffusion behavior and intermolecular interactions between bio-oil molecules and aged asphalt components. Experimental results show that bio-oils effectively restore the lost viscoelastic performance after long-term aging. An 8% dosage was determined as optimal, with rejuvenation efficiency decreasing in the order of SSO, SO, and PO. MD simulations clarify mechanisms by showing that soybean and palm oils have higher diffusion efficiency than sunflower oil, thus promoting the dispersion of asphaltene and resin. RDF shows that bio-oils enhance asphalt molecules’ short-range order via hydrogen bonds and van der Waals forces, which improves compatibility.

## 1. Introduction

Asphalt pavement is the main roadway stream for highways [1]. However, asphalt materials are prone to aging due to various environmental conditions. Oxidative aging is recognized to be the main factor that damages the asphalt molecular structure, changing its viscoelastic response, making it brittle and prone to cracking, which largely compromise the service performance of asphalt concrete, especially for cold regions [2,3,4].

In terms of asphalt rejuvenation, bio-oil is an effective and environmentally friendly material capable of restoring the performance of aged asphalt [5,6]. Existing research has explored the rejuvenation mechanisms and effectiveness of bio-oils from various perspectives. Some studies have demonstrated that various bio-oils can restore the viscoelastic performance of asphalt, with an optimum dosage typically between 5% and 12% [7,8,9,10]. It was also found that asphalt type, aging degree, and temperature significantly impact the rejuvenation efficiency [11,12].

To investigate the rejuvenation mechanism, the molecular dynamics (MD) simulation method was employed to analyze the compatibility and diffusion behavior between bio-oils and asphalt components [13]. Zhang et al. used molecular dynamics simulations to evaluate soybean oil as a rejuvenator for aged asphalt-binder, calculating diffusion coefficients and solubility parameters and finding that soybean oil had a high diffusion coefficient and good compatibility with asphalt components [14]. By combining the analysis of thermodynamic parameters, Ren et al. found that the degree of aging and temperature affects the compatibility between the rejuvenator and aged asphalt [15]. Shi et al. studied the regeneration mechanism of waste soybean oil (WSO) and functionalized SBS polymer on aged asphalt via molecular dynamics simulations [16]. Li et al. investigated the recovery performance of waste vegetable oil on aged asphalt; with the help of indicators like the radial distribution function (RDF) and the mean squared displacement (MSD), they discovered that waste vegetable oil significantly influences the diffusion of SARA fractions in asphalt [17]. Xu et al. studied the diffusion and interaction mechanisms of rejuvenators in virgin asphalt and aged asphalt, revealing the impact of rejuvenators on diffusion behavior [18]. However, a systematic comparative study on the rejuvenation efficiency and mechanisms of different types of bio-oils, especially for aged high-penetration asphalt, remains limited [19].

**Figure 1:**
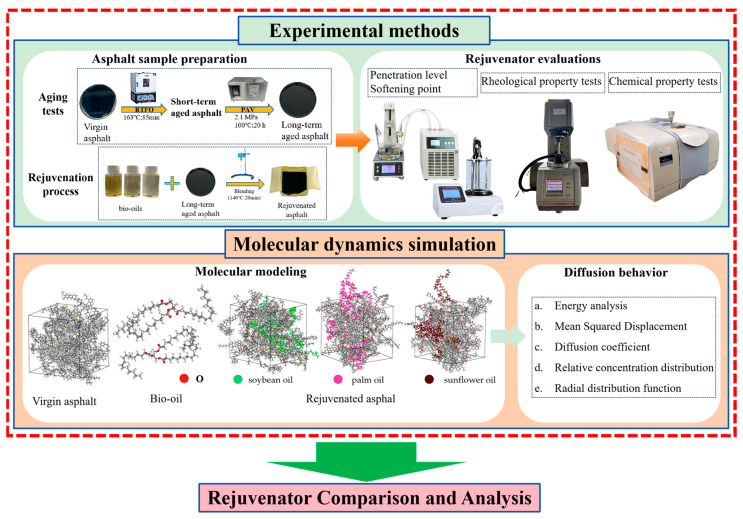
Research framework.

In this study, the rejuvenation effect of various bio-oils was investigated through experimental investigation and molecular dynamics simulation. As shown in Figure 1, PEN 90 asphalt was first aged and then rejuvenated with three different bio-oils, namely palm oil, soybean oil, and sunflower oil. Needle penetration, softening point, dynamic shear rheological test, and FTIR were carried to investigate the rejuvenation effect of the bio-oils. Additionally, the molecular dynamics simulation method was employed to investigate the rejuvenation mechanism. Finally, a bio-oil evaluation system was built to rank the bio-oils based on their overall performance in aged asphalt rejuvenation.

## 2. Materials and Experimental Methods

### 2.1. Asphalts and Rejuvenators

The asphalt utilized in this study is virgin PEN 90 asphalt (Hubei Guochuang Road Material Technology Co., Ltd., Wuhan, China), which is widely used as a high-penetration asphalt for pavement engineering for cold regions in China [20]. The measured physical properties are presented in Table 1. The virgin asphalt, short-term aged asphalt, and long-term aged asphalt are, respectively, referred to as VA, SAA, and LAA.

**Figure 2:**
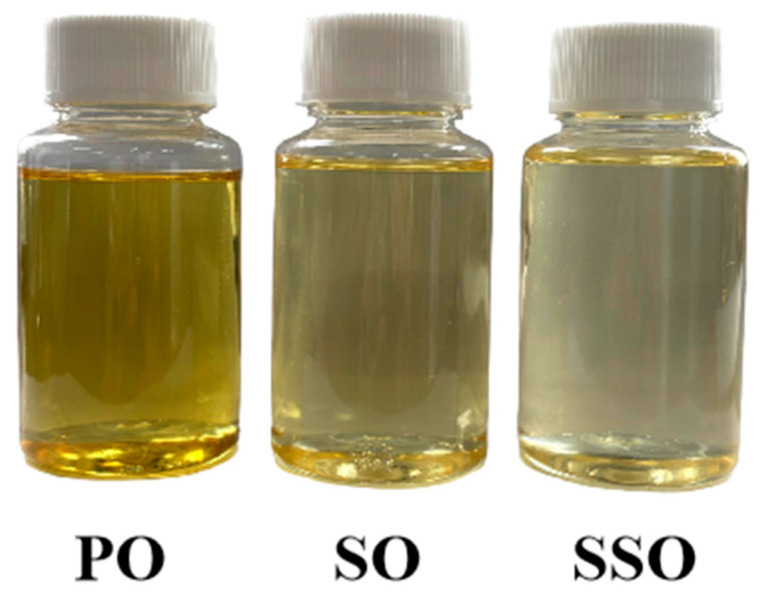
The appearance of the three bio-oils.

Figure 2 shows the image of three different bio-oil rejuvenators. Palm oil is a transparent yellow liquid, while soybean oil and sunflower oil have lighter colors. The palm oil, soybean oil, and sunflower oil are, respectively, referred to as PO, SO, and SSO.

### 2.2. Preparation of Aged and Rejuvenated Asphalt

Asphalt samples were first aged through standard Rolling Thin Film Oven (RTFO) and Pressure Aging Vessel (PAV) methods following ASTM-D2872 [21] and ASTM-D6521 [22], respectively, representing a real road aging level after 5 to 10 years of service. The virgin asphalt, short-term aged asphalt, and long-term aged asphalt are designated as VA, SAA, and LAA, respectively.

**Figure 3:**
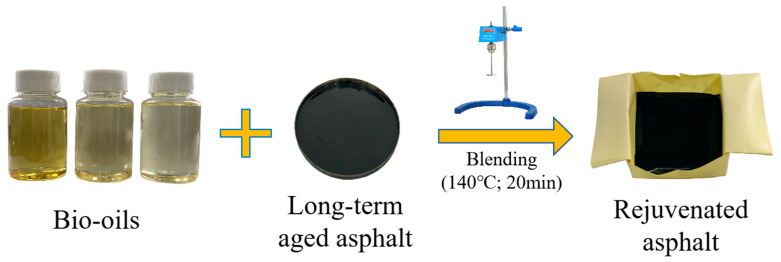
The procedure of aged asphalt rejuvenation.

Rejuvenated asphalt was prepared by mixing the long-term aged asphalt with bio-oil. The rejuvenation process is illustrated in Figure 3. In this procedure, the long-term aged asphalt samples were loaded into an aluminum tray. Bio-oil rejuvenators were then added to the asphalt samples at the dosages of 2 wt%, 4 wt%, 6 wt%, and 8 wt%. The samples were manually homogenized with a glass rod, followed by mechanical blending at 300 rpm for 5 min using a high-shear mixer and at a temperature of 140 °C for 20 min, thus acquiring the rejuvenated asphalt.

### 2.3. Experimental Methods

#### 2.3.1. Penetration Level and Softening Point

The performance of asphalt pavement is significantly influenced by the physical properties of the asphalt binder. Consequently, in this study, the determination of optimal bio-oil dosages for rejuvenating aged asphalt primarily relies on key physical properties, specifically penetration level and softening point. These properties were measured according to ASTM-D5 [23] and ASTM-D36 [24], respectively.

#### 2.3.2. Rheological Property Tests

The rheological properties of asphalt samples were investigated using a Dynamic Shear Rheometer (DSR) MCR102 (Anton Paar, Graz, Austria). A temperature sweep program, conducted at a frequency of 10 Hz, was used to determine the complex shear modulus (G*), phase angle (δ), and rutting parameter (G*/sin δ) of the samples across a temperature range of 30 °C to 80 °C. These measurements aimed to elucidate the changes in viscoelastic behavior and high-temperature performance of the asphalt during aging and rejuvenation processes [25]. The tests utilized 25 mm parallel plates with sample thickness maintained at 1 mm, under a strain-controlled mode with an applied strain of 0.12.

#### 2.3.3. Chemical Property Tests

Fourier Transform Infrared Spectroscopy (FTIR) tests were conducted on asphalt and three types of oils. The aging of asphalt induces chemical structural alterations, which are manifested by the increase in carbonyl groups (C=O) and sulfoxide groups (S=O) [26]. Thus, characteristic functional groups such as carbonyl and sulfoxide groups were selected for analysis in the Fourier Transform Infrared Spectroscopy (FTIR) scanning results. The carbonyl index (*I_C_*) and sulfoxide index (*I_s_*) were derived to characterize the impact of aging and rejuvenation on the internal components and molecular structure of asphalt [27,28]. To comprehensively evaluate the overall oxidation level of asphalt, we have developed a comprehensive index. The comprehensive index is defined as the sum of the carbonyl index and the sulfoxide index. This index can characterize the oxidation state more comprehensively compared with a single index. As expected, the higher the value of the comprehensive index, the higher the corresponding oxidation degree. Each asphalt sample and each type of oil was scanned 32 times, with a testing resolution of 4 cm^−1^ and a testing range of 500–4000 cm^−1^.

(1) $$I_{C}=\frac{A_{1696}}{\sum A}$$

(2) $$I_{S}=\frac{A_{1030}}{\sum A}$$

(3) $$\sum A=A_{725}+A_{746}+A_{810}+A_{865}+A_{1030}+A_{1373}+A_{1456}+A_{1601}+A_{1696}+A_{2853}+A_{2921}$$

where $A_{i}$ is the peak area at the corresponding wavenumber of the characteristic functional group; $I_{C}$ is the carbonyl index and $I_{s}$ is the sulfoxide index.

### 2.4. The Radar Chart Method

In the comprehensive performance evaluation of bio-oil-rejuvenated asphalt, the radar chart method was adopted as an intuitive and efficient analytical approach. Focusing on bio-oil-rejuvenated asphalt as the core research subject, this method generates radar charts via data visualization tools, enabling the clear presentation of asphalt performance across multiple key performance indicators. The specific implementation procedures are as follows:

1. Confirm the bio-oil-rejuvenated asphalts for analysis and define the corresponding key performance indicators;
2. Determine the positive/negative attributes of each indicator, then process the raw data using the normalization formula to unify the order of magnitude of different indicators;
3. Import the normalized data into the data visualization tool for radar chart generation, which provides visual support for the subsequent quantitative analysis of comprehensive performance and the evaluation of performance superiority.

## 3. Experimental Results and Discussion

### 3.1. Physical Properties

**Figure 4:**
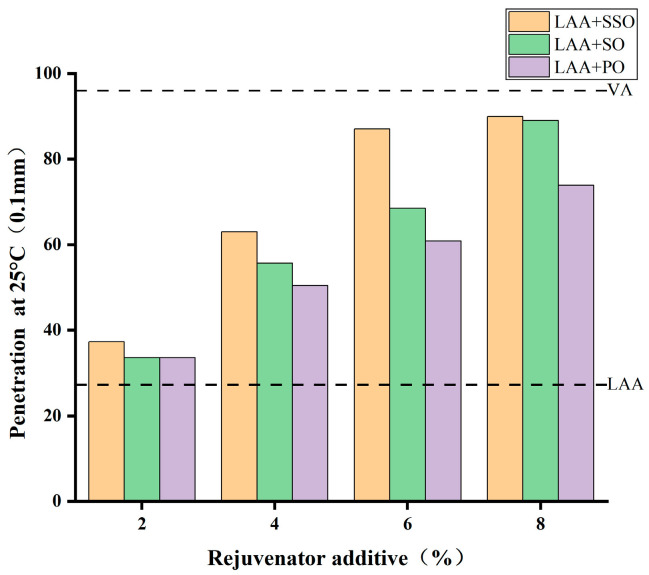
Penetration of rejuvenated asphalt with the three bio-oils at different dosages.

Figure 4 shows the penetration results of the rejuvenated asphalt, in which SSO-rejuvenated asphalt, SO-rejuvenated asphalt, and PO-rejuvenated asphalt are denoted as LAA+SSO, LAA+SO, and LAA+PO, respectively. It shows that the penetration of virgin asphalt decreases from 96 (0.1 mm) to 27.3 (0.1 mm) after long-term aging. When the dosage of the three bio-oils is 2%, the reduction in penetration of aged asphalt is insignificant. As the dosage increases, a recovery trend gradually emerges, with the penetration increasing gradually, indicating that the addition of these three bio-oils can restore the penetration of aged asphalt. At an 8% dosage, the penetration of the rejuvenate asphalt approaches that of the virgin asphalt. In terms of restoring the penetration of aged asphalt, SSO and SO exhibit better effects than PO.

**Figure 5:**
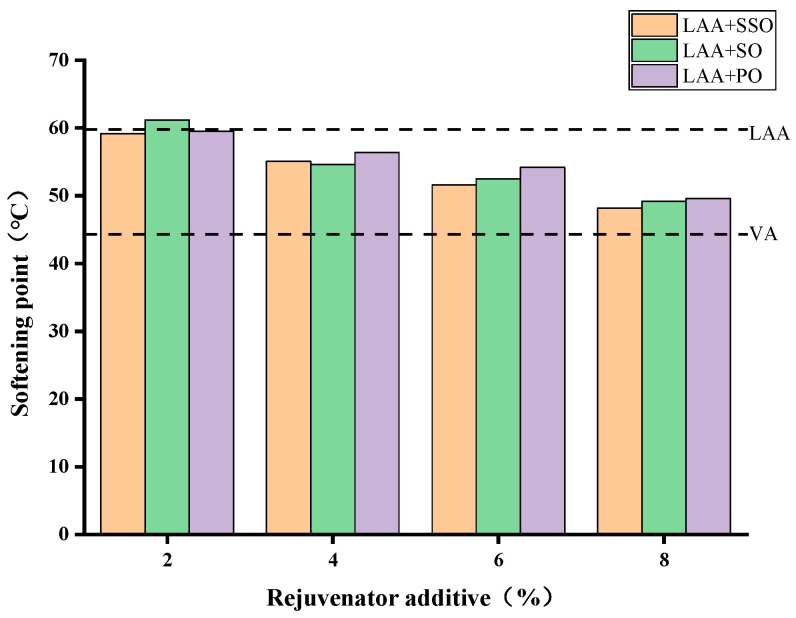
Softening point of rejuvenated asphalt with the three bio-oils at different dosages.

Figure 5 presents the softening point results of the asphalt samples. The softening point of long-term aged asphalt increases from 44.3 °C to 59.8 °C compared with virgin asphalt. Adding bio-oil gradually restores the softening point of asphalt, with more pronounced effects as the dosage increases. At an 8% dosage, the softening point of rejuvenated asphalt approaches that of virgin asphalt. However, the three bio-oils show little difference in their effectiveness in reducing the softening point of asphalt.

### 3.2. Rheological Properties

**Figure 6:**
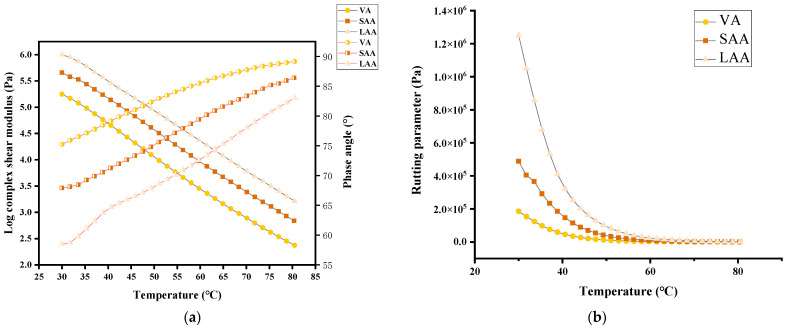

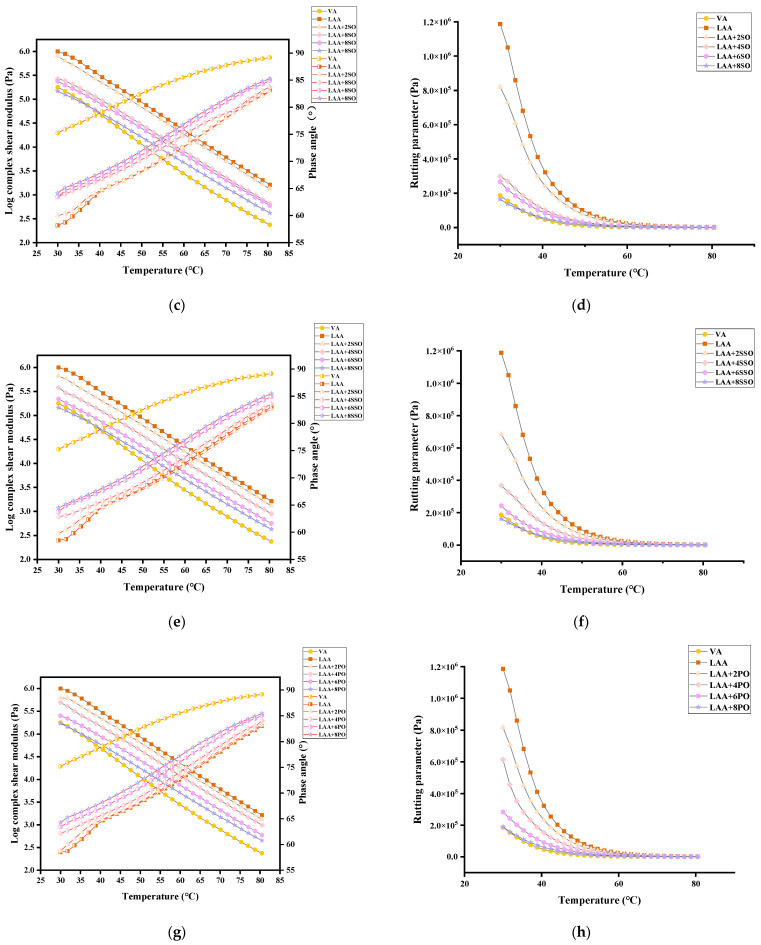
Complex shear modulus, phase angle, and rutting parameter for virgin asphalt, aged asphalt, and rejuvenated asphalt: (**a**,**b**) are the complex shear modulus, phase angle, and rutting parameter for virgin asphalt and aged asphalt; (**c**,**d**) are the complex shear modulus, phase angle, and rutting parameter for SO-rejuvenated asphalt; (**e**,**f**) are the complex shear modulus, phase angle, and rutting parameter for SSO-rejuvenated asphalt; and (**g**,**h**) are the complex shear modulus, phase angle, and rutting parameter for PO-rejuvenated asphalt.

Figure 6 shows the complex shear modulus, phase angle, and rutting parameter of asphalt with different aging degrees and rejuvenated asphalt. The complex shear modulus reflects asphalt’s ability to resist shear strain, the phase angle characterizes the delay in mechanical response between the viscous and elastic components of asphalt, and the rutting parameter represents the capacity of asphalt pavement to resist permanent deformation [29,30].

Figure 6a,b show that aging increases the complex shear modulus and rutting parameter of asphalt samples and decreases the phase angle. In the lower temperature range, the elastic modulus of aged asphalt is significantly higher, leading to an increase in the rutting parameter. However, as the temperature rises, the differences in viscoelastic properties among asphalts with different aging degrees gradually narrow, resulting in converging performance.

The results in Figure 6c–h indicate that all three types of bio-oil can partially restore the performance of long-term aged asphalt: the complex shear modulus (G*) and rutting parameter (G*/sin δ) decrease, while the phase angle (δ) increases. Notably, as the bio-oil content increases, the rutting parameter decreases gradually and eventually reaches a level close to that of the virgin asphalt, which indicates that its high-temperature rutting resistance is restored to the state of the virgin asphalt.

When the content of the three bio-oils reaches 8%, the complex shear modulus (G*), phase angle (δ), and rutting parameter (G*/sin δ) of the rejuvenated asphalt are all restored to levels similar to those of the virgin asphalt. This trend is consistent with the penetration and softening point results. It is also found that the increase in the phase angle of SO-rejuvenated asphalt is significantly smaller than that of the SSO- and PO-rejuvenated asphalt, which indicates that the restoration efficiency of SO on the molecules of aged asphalt is relatively weak. With the increase in the content of the three bio-oils, the rutting parameter decreases significantly in the lower test temperature range (40–60 °C), while the decreasing trend gradually flattens out in the high-temperature range (>60 °C). The rutting parameter (G*/sin δ) of the rejuvenated asphalt shows a systematic decreasing trend, which indicates that the bio-oil content needs to be controlled within a reasonable threshold.

It is worth noting that except for the condition with 8% bio-oil content, the rutting parameter values of rejuvenated asphalt with other contents are all higher than that of the virgin asphalt, proving that it still maintains excellent high-temperature stability. Based on a comprehensive analysis of rheological behavior, the optimal dosage for the three bio-oils should be set at 8%.

### 3.3. Chemical Properties

**Figure 7:**
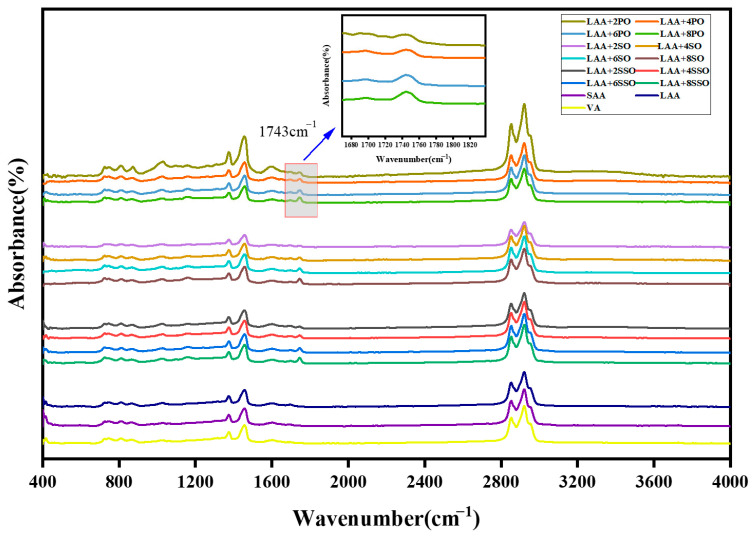
FTIR spectra of rejuvenated asphalt and aged asphalt.

Figure 7 presents the FTIR spectra of virgin, aged, and rejuvenated asphalt. The aged asphalt’s spectral changes mainly occur in characteristic functional group peak regions: the peak areas of sulfoxide groups (1030 cm^−1^) and carbonyl groups (1696 cm^−1^) increase. Figure 7 shows that rejuvenated asphalt exhibits a new characteristic absorption peak at 1743 cm^−1^, while long-term aged asphalt has no obvious response in this waveband. Notably, this 1743 cm^−1^ peak corresponds to the stretching vibration of C=O in ester groups, matching the functional group characteristics of the three bio-oils. This proves that bio-oil successfully introduces active ester components into aged asphalt. Additionally, the peak area ratio is positively correlated with bio-oil content: a significant increase is observed when the content rises from 2% to 8%.

**Figure 8:**
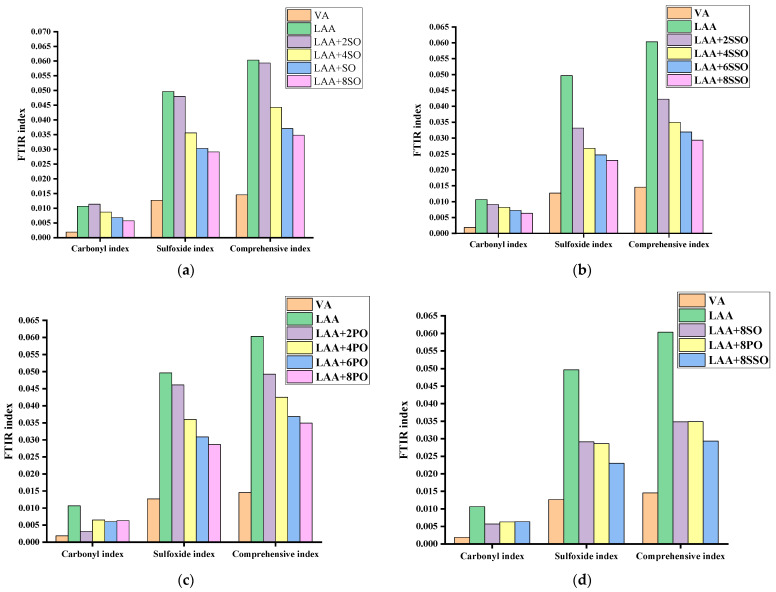
FTIR index of rejuvenated asphalts. (**a**) FTIR index of soybean oil; (**b**) FTIR index of sunflower oil; (**c**) FTIR index of palm oil; and (**d**) FTIR index of rejuvenated asphalt at the dosage with 8%.

Figure 8 shows the FTIR indices of aged asphalt incorporated with soybean oil, palm oil, and sunflower oil, respectively. It is found that the FTIR indices of rejuvenated asphalt gradually decrease with the increase in bio-oil dosage, indicating that all three bio-oils can alleviate the aging of aged asphalt. This may be attributed to either physical dilution of aging-induced carbonyl (C=O) and sulfoxide (S=O) groups, resulting in a direct decrease in the concentration of polar groups per unit volume and subsequent reduction in FTIR peak areas, or the reduction in sulfoxide groups (S=O) by active components in bio-oils (such as aldehyde groups and unsaturated bonds), which weakens the sulfoxide peak intensity at 1030 cm^−1^. Additionally, fatty acids in vegetable oils may undergo esterification reactions with carboxylic acids (R-COOH) generated from asphalt oxidation, dispersing the original carbonyl peak area [31].

When the bio-oil dosage reaches 8%, the FTIR characteristic indices (carbonyl index CI and sulfoxide index SI) of the three rejuvenated asphalts all recover to levels similar to those of virgin asphalt. Figure 8d compares the rejuvenation efficiencies of SSO (sunflower oil), SO (soybean oil), and PO (palm oil) at this dosage. The results show that the FTIR indices of the rejuvenated asphalts follow the following order: SSO-rejuvenated asphalt < SO-rejuvenated asphalt < PO-rejuvenated asphalt. This pattern indicates that the rejuvenation efficiency of bio-oils for the chemical components of aged asphalt decreases in the order of SSO, SO, and PO. Notably, the rejuvenation effects of SO and PO are not significantly different, while SSO exhibits the optimal functional group repair capability.

**Figure 9:**
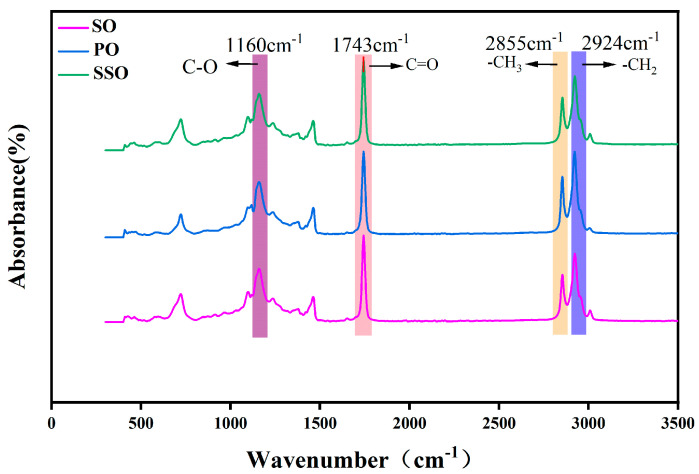
FTIR of three bio-oils.

The FTIR spectra of the three bio-oils are shown in Figure 9. In the FTIR spectra of the three bio-oils, the absorption at 750 cm^−1^ corresponds to the out-of-plane bending vibration of C-H in the benzene ring, and the absorption at 1160 cm^−1^ corresponds to the asymmetric stretching vibration of C-O in the ester group (C-O-C). The absorption at 1450 cm^−1^ may overlap with the C=C skeletal vibration of the benzene ring, reflecting the presence of aromatic compounds and the characteristics of aromatic structures in bio-oils. The absorption at 1743 cm^−1^ corresponds to the stretching vibration of C=O in the ester group, and the absorption peaks at 2855 cm^−1^ and 2924 cm^−1^ correspond to the stretching vibrations of methyl and methylene groups [32].

## 4. Molecular Dynamics Simulation

### 4.1. Molecular Models of Asphalt

**Figure 10:**
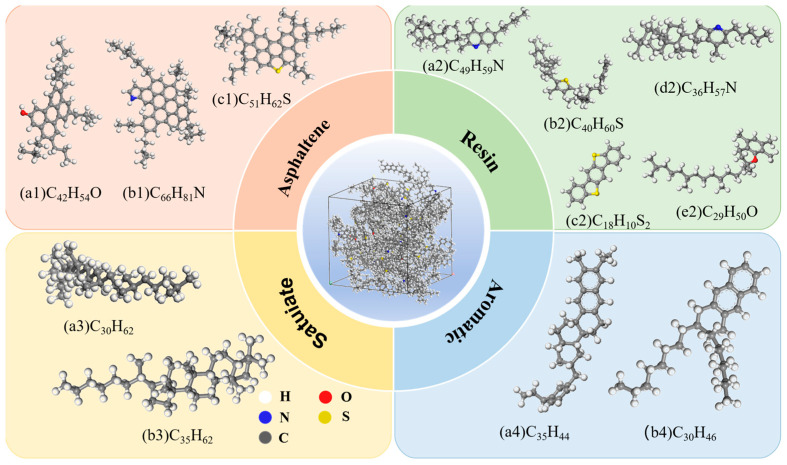
Twelve representative molecular models for asphalt components.

Molecular Dynamics Simulation (MDS) aims to simulate the dynamic behavior of molecular systems. It can particularly illustrate the mechanisms during asphalt aging and rejuvenation [33]. In this study, the virgin asphalt molecular model was constructed using the four-component twelve-molecule model proposed by D.D. Li et al., via Materials Studio 2020 [34]. The four-component twelve-molecule model is shown in Figure 10.

First, the model underwent geometric optimization via the COMPASSⅢ force field and Smart algorithm (minimum iterations: 10,000) to reach the lowest energy state.

The virgin asphalt molecular model was built using the Amorphous Cell module: optimized molecules and their quantities were selected to construct an amorphous cell with an initial density of 0.8 g/cm^3^. The aforementioned force field and algorithm were adopted, where the Ewald method was used to calculate the system’s electrostatic energy and the Atom-based method was employed to compute the van der Waals energy (cutoff radius: 12.5 Å).

**Figure 11:**
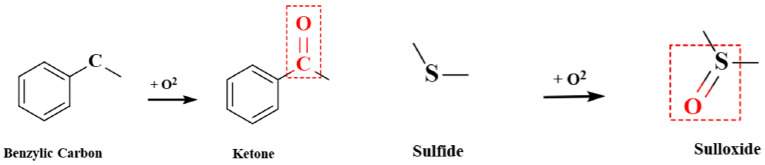
Major oxidation reactions and products in asphalt.

The preliminarily established asphalt model was optimized with identical parameters. Asphalt aging, mainly caused by carbonyl and sulfoxide formation, was modeled by modifying corresponding groups in the virgin asphalt model, with components and molecular counts unchanged. The major oxidation reactions and products in asphalt are shown in Figure 11.

**Figure 12:**
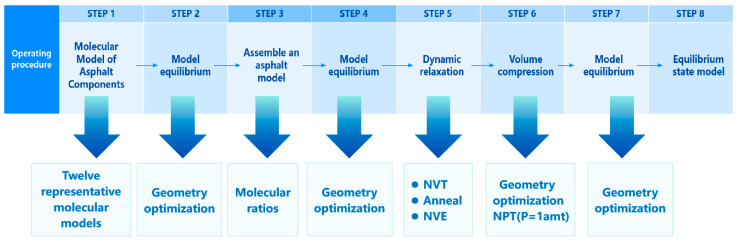
The dynamic equilibrium operating procedure of the asphalt model.

**Figure 13:**
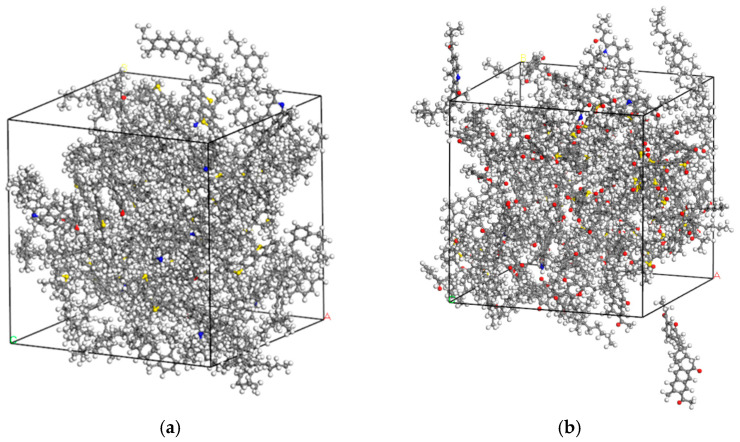
Virgin and long-term aged asphalt models. (**a**) Virgin asphalt model; (**b**) Long-term aged asphalt model.

The dynamic equilibrium operating procedure was completed through eight steps, which is illustrated in Figure 12. The constructed virgin and aged asphalt models are shown in Figure 13.

**Figure 14:**
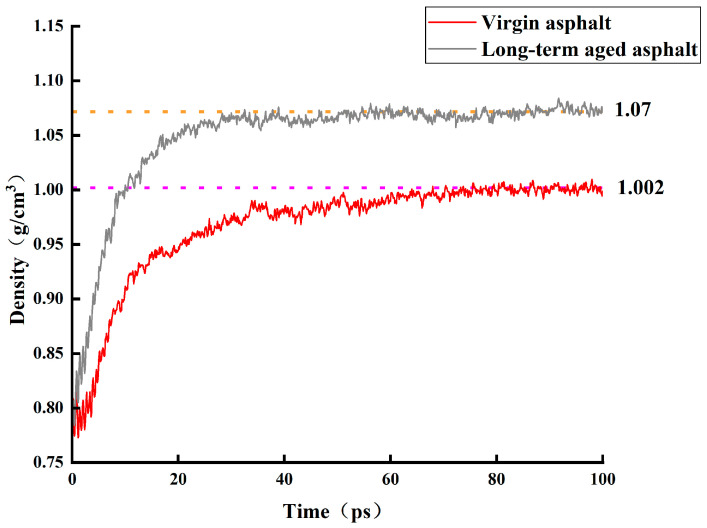
Densities of virgin and long-term aged asphalt models.

The density acquired from the MDS characterizes the model’s mechanical properties and simulation accuracy and validates the model’s effectiveness. As shown in Figure 14, the virgin asphalt density gradually increases over time steps, stabilizing at approximately 1.002 g/cm^3^ when the time step reaches 100 ps; the aged asphalt density is slightly higher, stabilizing at approximately 1.07 g/cm^3^. Both simulated densities fall within the 5% allowable fluctuation range of experimentally measured asphalt density, demonstrating a reasonably constructed asphalt model.

### 4.2. Molecular Models of Bio-Oils

**Figure 15:**
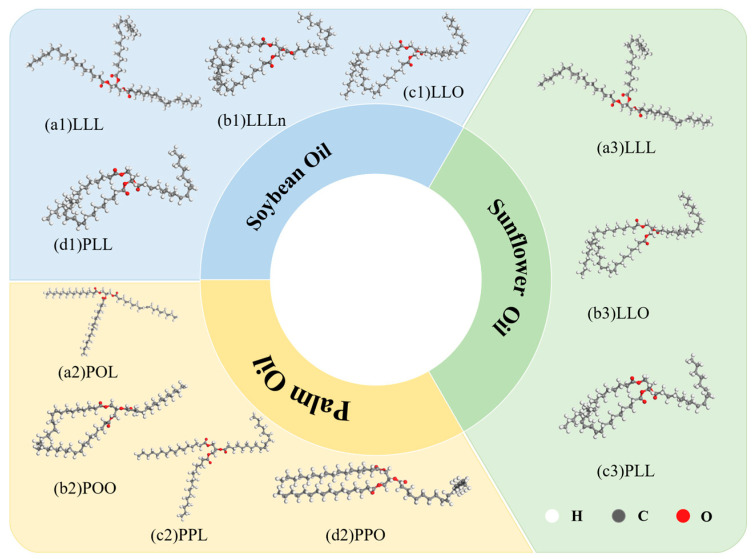
Molecular models of three bio-oils.

As vegetable oils, the three bio-oils are mainly composed of triglycerides (TG), with a content as high as 95% [35]. The composition of triglycerides is related to the types of fatty acids contained. Combined with the Fourier Transform Infrared Spectroscopy (FTIR) tests of three vegetable oils and the relevant research by Wei Wei [36], it was found that the main triglyceride components of soybean oil are LLL, LLLn, LLO, and PLL; the main triglyceride components of sunflower oil are LLL, LLO, and PLL; and the main triglyceride components of palm oil are PPO, POO, POL, and PPL. Based on this, the molecular models of the three vegetable oils were established, as shown in Figure 15.

### 4.3. Molecular Models of Rejuvenated Asphalts

When performing molecular dynamics calculations, considering the size of the calculation model, the content of bio-oil molecules in the rejuvenated asphalt model was set to approximately 12% of the mass of the aged asphalt, as shown in Table 2.

**Figure 16:**
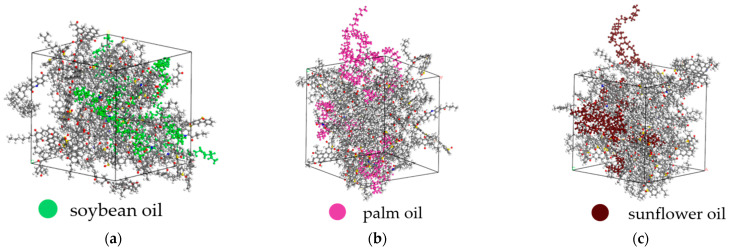
(**a**) Soybean oil-rejuvenated asphalt model, (**b**) palm oil-rejuvenated asphalt model, and (**c**) sunflower oil-rejuvenated asphalt model.

When establishing the rejuvenated asphalt molecular model, to make the mixing process of bio-oil molecules and aged asphalt molecules closer to the actual situation, the optimized bio-oil molecules and aged asphalt molecules were moved into a unit cell with an initial concentration of 0.8 g/cm^3^. Then, model equilibration, relaxation, and volume compression were carried out to fully mix the bio-oil molecules and aged asphalt molecules, making the volume close to the actual state. Finally, an equilibrium state model was obtained, with parameters and calculation step sizes set the same as those in the previous section. The three rejuvenated asphalt molecular models are shown in Figure 16.

### 4.4. Diffusion Behavior

#### 4.4.1. Energy Analysis

**Figure 17:**
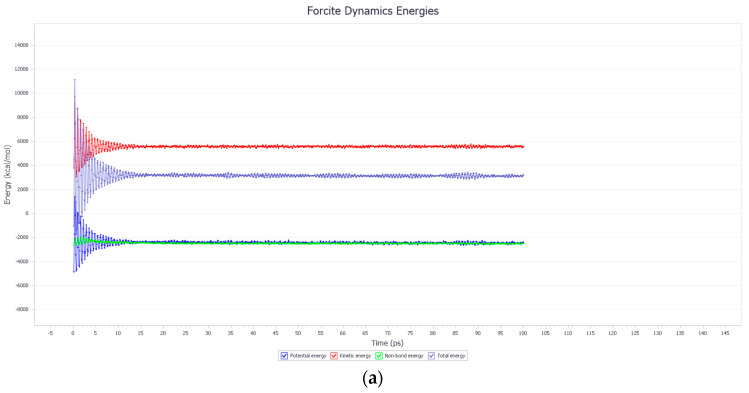

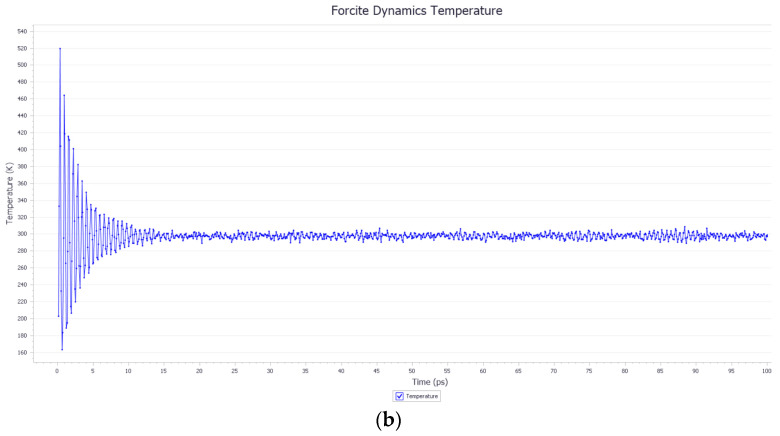
(**a**) The energy change in the soybean oil-rejuvenated asphalt model and (**b**) the temperature change in the soybean oil-rejuvenated asphalt model.

In molecular dynamics simulations, the diffusion of asphalt is a spontaneous process. The change in the total energy of the system is primarily caused by fluctuations in potential energy and kinetic energy. Since chemical bonds are relatively stable, the change in bond energy (internal energy) is usually minimal and can be ignored. Therefore, the main energy changes are reflected in potential energy and kinetic energy. When the system is heated, the energy distribution changes accordingly. For the rejuvenated asphalt model, the molecules within the model continuously diffuse until a stable state of dynamic equilibrium is achieved. Taking the soybean oil-rejuvenated asphalt molecular model as an example, its energy gradually stabilizes over time. The variation process is illustrated in Figure 17.

#### 4.4.2. Mean Squared Displacement

During diffusion simulation, intermolecular interactions cause molecules to interpenetrate and eventually reach equilibrium. The mean squared displacement (MSD), a measure of the average squared displacement of molecules, directly correlates with molecular mobility. It can thus evaluate the efficiency of energy (heat) transfer in the system and describe the diffusion process [37].

**Figure 18:**
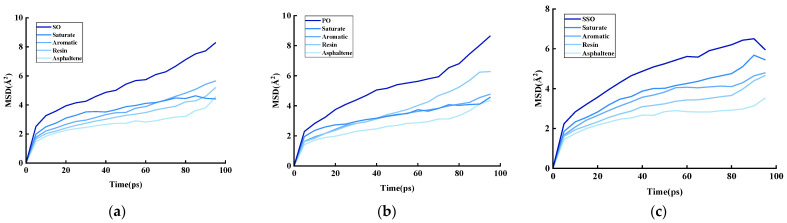
(**a**) Mean squared displacement of soybean oil-rejuvenated asphalt, (**b**) mean squared displacement of palm oil-rejuvenated asphalt, and (**c**) mean squared displacement of sunflower oil-rejuvenated asphalt.

At a system temperature of 298 K, the diffusion behavior of different vegetable oils in asphalt was studied. The rejuvenated asphalt models constructed earlier were run in the NPT ensemble for 100 ps to achieve equilibrium. The particle motion was analyzed using the Analysis command in the Forcite module to obtain MSD curves. The MSD variations in the three bio-oils at 298 K are shown in Figure 18.

It is known that the slope of the mean squared displacement (MSD)–time curve correlates with the diffusion coefficient, meaning the variation in the MSD curve directly reflects the molecular diffusion rate. As shown in the figure, the MSD curves of the three models all exhibit a smooth upward trend, indicating that the constructed models possess notable fluidity.

Given the random nature of diffusion behavior in the simulation, the diffusion process is analyzed based on curve characteristics: during the 0–10 ps interval, aged asphalt and bio-oil undergo rapid diffusion upon contact. The high initial slope of the curve stems from the fast, irregular motion of particles within the system. After 10 ps, molecules diffuse into the interior to fill micro-voids, gradually reaching a relatively stable state. The curve slope then stabilizes, demonstrating a linear positive correlation between MSD and time. This behavior aligns with the equilibrium state in molecular simulations. Comparing MSD values of the three bio-oils reveals that soybean oil and palm oil exhibit superior diffusion efficiency compared to sunflower oil.

#### 4.4.3. Diffusion Coefficient

The diffusion coefficient is a key parameter for measuring the speed of substance diffusion, which can more directly reflect the relative diffusion rates of different substances. The diffusion coefficient can be calculated by analyzing the variation law of the mean squared displacement (MSD) with time and using the relationship between them. The MSD can be obtained directly from the particle positions in a molecular dynamics simulation. If r(t) is the position at time t, and r(t + Δt) the position an interval Δt later, the squared displacement of the particle during that interval is [(r(t + Δt) − r(t)]^2^. In an equilibrium ensemble, the average squared displacement must be independent of the time t, and can be averaged out, leaving the mean squared displacement over an interval Δt [38]:

(4) $$\mathrm{MSD}(\Delta t)=\frac{1}{\tau -\Delta t}\int_{0}^{\tau -\Delta t}\left[r(t-\Delta t)-r(t\right){]}^{2}\;\mathrm{dt}=\langle {\left[(r(t-\Delta t)-r(t)\right]}^{2}\rangle$$

where *τ* is the total simulation time.

If the particle is bound, the MSD levels off to a constant. If the particle is diffusing, the MSD becomes linear in time (diffusive regime); the slope defines the diffusion coefficient *D*, according to the following Einstein equation:

(5) $$D=\frac{1}{6}\underset{\Delta t\to 0}{\lim}\frac{d\mathrm{MSD}}{d\Delta t}\approx \frac{1}{6}K_{\mathrm{MSD}}$$

where K_MSD_ is the slope of the mean squared displacement (MSD)–time curve.

**Figure 19:**
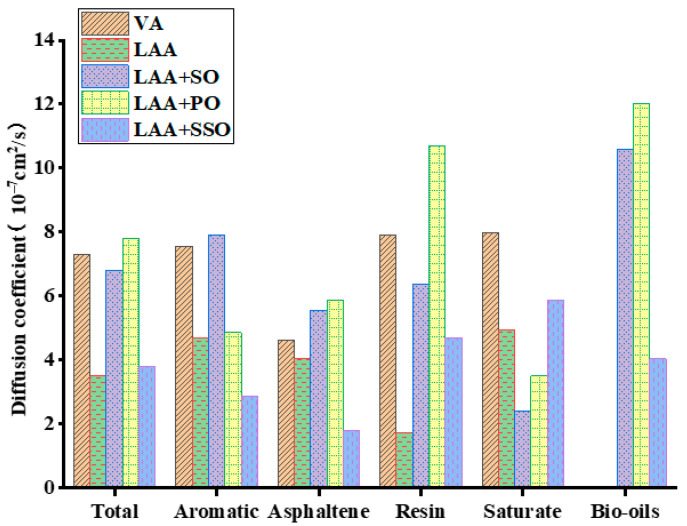
Diffusion coefficients of four asphalt components and bio-oils.

Based on the mean squared displacement (MSD) curves, the slopes of the MSD–time curves for virgin asphalt, aged asphalt, and rejuvenated asphalt were obtained through fitting. The parameters in fitting curves of different asphalt models are shown in Table 3. According to the relationship between the diffusion coefficient and the slope of the MSD–time curve, the diffusion coefficients can be calculated. The diffusion coefficients of each rejuvenated asphalt model are shown in Figure 19.

As shown in the figure above, overall, the diffusion coefficient of long-term aged asphalt is the lowest. With the addition of vegetable oils, the diffusion coefficient increases. Specifically, the diffusion coefficients of aged asphalt incorporated with soybean oil and palm oil increase significantly, exceeding or approaching the level of virgin asphalt. For the four components of asphalt, soybean oil and palm oil exhibit better effects in restoring the diffusion of asphaltenes, saturates, and resins, while sunflower oil shows poorer performance. Notably, among vegetable oil components, soybean oil and palm oil have larger diffusion coefficients, indicating their superior diffusion effects and better capability to improve the properties of aged asphalt. These data suggest that soybean oil and palm oil can effectively enhance the properties of aged asphalt and improve its diffusion capacity in various components, which is of great significance for upgrading the overall performance of asphalt materials.

#### 4.4.4. Relative Concentration

**Figure 20:**
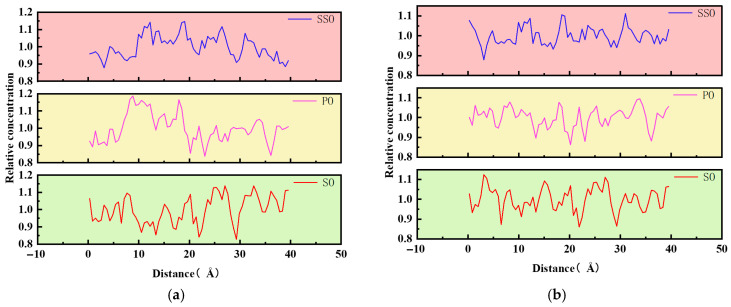
(**a**) The relative concentration distribution of asphalt molecules. (**b**) The relative concentration distribution of bio-oil molecules.

Diffusion movement mainly originates from the concentration gradient or other acting forces between two-phase substances, manifested as the spatial migration of substances. In a specific system, for quantitative analysis, the constructed unit cell can be homogeneously divided into several sub-regions. According to the ratio of the number of molecules to the occupied volume in each sub-region, its local concentration is determined. The ratio of the local concentration to the average concentration of the entire unit cell is defined as the relative concentration. Finally, the calculated relative concentration data are subjected to fitting processing. The relative concentrations of asphalt molecules and bio-oil molecules in the model unit cell of rejuvenated asphalt are shown in Figure 20.

By comparing the relative concentration change curves of asphalt molecules in the three models, it can be seen that the volatility order is SO > PO > SSO. That is, in the rejuvenated asphalt models with soybean oil and palm oil added, the discreteness of displacement of old and new asphalt molecules is greater, indicating that the addition of soybean oil and palm oil has a better promoting effect on the diffusion of aged asphalt. The above analysis results show to a certain extent that the addition of bio-oil can promote the mutual movement of asphalt molecules.

#### 4.4.5. Radial Distribution Function

The radial distribution function (RDF) is used to analyze the microscopic distribution of particles. It represents the spatial probability distribution of particle B in the shell layer from r to r + dr around the central particle A. RDF helps study the packing of particles: for long-range interactions, the RDF curve flattens gradually and approaches a constant value as distance increases. When the distance between particle B and central particle A is sufficient, RDF tends to 1 [39].

**Figure 21:**
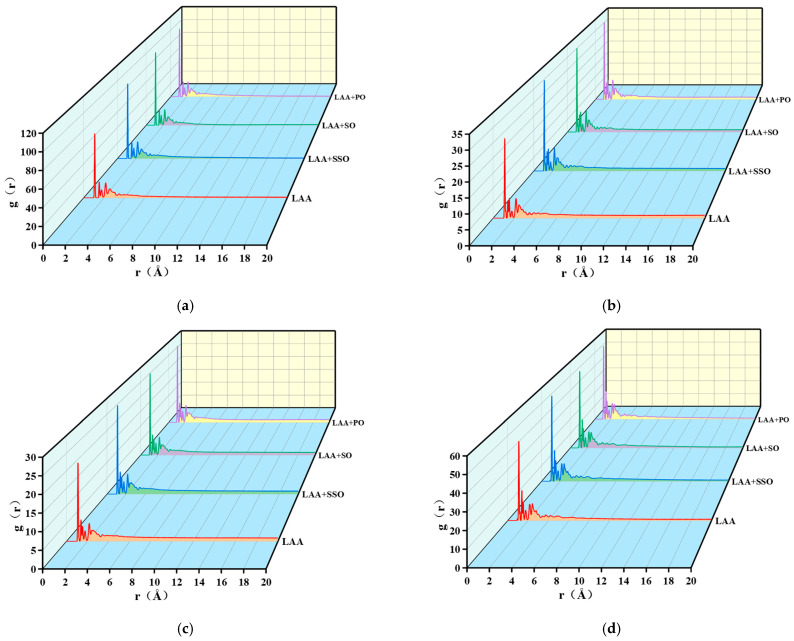
The RDF for the four components of asphalt. (**a**) The RDF for the saturate, (**b**) the RDF for the aromatic, (**c**) the RDF for the resin and (**d**) the RDF for the asphaltene.

Figure 21 shows the relative distance distributions (RDF) of the four components in asphalt with different bio-oils. For r < 5 Å (angstroms), all four asphalt molecular models exhibit three distinct peaks. When r > 5 Å, the g(r) value approaches 1, indicating an overall disordered state in the asphalt. Collectively, the RDF characteristics of the four asphalt models show short-range order and long-range disorder, consistent with the RDF features of amorphous materials.

With the addition of bio-oils, the peak positions remain essentially unchanged, suggesting that bio-oil addition does not affect the inter-component distances. Additionally, the overall shapes and trends of the four asphalt components are largely similar. Notably, bio-oils enhance the height of each peak. For saturates, the first-peak intensities for PO, SO, SSO, and LAA are 102.044%, 101.544%, 96.287%, and 72.993%, respectively. For aromatics, the first-peak intensities are 33.786%, 33.685%, 33.248%, and 26.463%. For resins, they are 28.60%, 28.04%, 26.37%, and 22.21%. For asphaltenes, the first-peak intensities are 52.962%, 51.4479%, 54.3964%, and 45.938%.

The increase in the first peak indicates that bio-oils enhance molecular densification in the entire asphalt system, with soybean oil and palm oil demonstrating more pronounced effects than sunflower oil. The radial distribution functions (RDFs) of all asphalt components peak between 1 and 4 angstroms (Å), confirming that hydrogen bonds and van der Waals forces promote ordered arrangement, higher aggregation, and stronger interactions among asphalt components at this distance. Bio-oils form stable three-dimensional network structures with asphalt molecules through physical crosslinking, which not only facilitates the formation of a stable composite structure but also improves compatibility between bio-oils and asphalt. Additionally, the adsorption of bio-oils onto asphalt molecules increases packing density, thereby enhancing the orderliness and structural stability of the asphalt.

## 5. Bio Rejuvenator Ranking

Through the previous simulations and analyses, the optimal dosage of bio-oil in rejuvenated asphalt was determined to be 8%. This study selected six parameters as design parameters: diffusion coefficient, penetration, softening point, FTIR index, rutting parameter at 80 °C, and phase angle at 80 °C. These parameters were normalized, where larger values for the diffusion coefficient, penetration, and phase angle are preferred (positive indicators), while smaller values for the FTIR index, softening point, and rutting parameter at 80 °C are preferred (negative indicators). This is related to the performance of asphalt. The calculation formula for positive indicators is shown in Equation (6), and that for negative indicators is shown in Equation (7). The original inputs for the five parameters are shown in Table 4.

(6) $$x^{\prime }=\frac{x-\min(X)}{\max(X)-\min(X)}$$

(7) $$x^{\prime }=1-\frac{x-\min(X)}{\max(X)-\min(X)}$$

**Figure 22:**
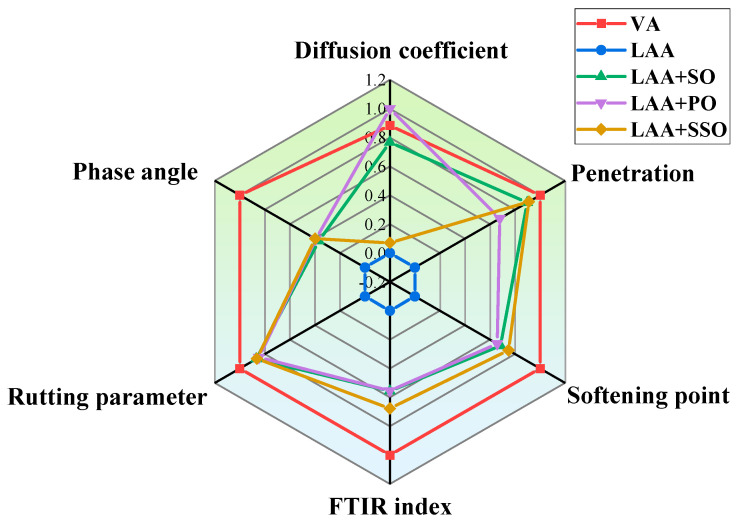
The performance comparison of three types of rejuvenated asphalt.

Figure 22 is a radar chart for comparing the multi-performance parameters of asphalt materials. Through six key parameters, it can intuitively present the performance differences in different asphalt samples (VA, LAA, and LAA with different rejuvenators added).

All the parameter lines of LAA are clearly close to the inside, and its performance is the weakest. LAA+SO has a significant improvement in terms of diffusion coefficient, penetration, and softening point. Its viscoelasticity is relatively balanced, which has an optimizing effect on high-temperature stability. LAA+SSO is relatively prominent in terms of rutting parameter, softening point, penetration, and FTIR index, and has an obvious optimization in terms of high-temperature deformation resistance, but its diffusion effect is slightly insufficient. LAA+PO has the best performance in terms of diffusion coefficient, and there is not much difference in terms of rutting parameter and phase anglecompared with the other two rejuvenated asphalts [40].

Through these six core parameters, the performance differences in the three bio-oils can be clearly seen, which can provide support for the subsequent research and development of compound bio-oil rejuvenators.

## 6. Conclusions and Recommendations

In this study, molecular models of virgin asphalt, aged asphalt, and rejuvenated asphalt were established using molecular dynamics simulation software, and the accuracy of the models was verified by density. Subsequently, the diffusion behavior analysis of three types of rejuvenated asphalts, namely LAA+SO, LAA+PO, and LTA+SSO, was carried out through molecular dynamics simulation. Their physical properties were tested, the FTIR spectra of the samples were tested, and the FTIR indices were analyzed. The rheological properties of the samples were tested by DSR experiments. The main conclusions finally obtained are as follows:

(1) Asphalt physical property tests show that aging raises softening point and markedly lowers penetration. Bio-oil helps both recover, with better effects at higher dosages. The three bio-oils perform similarly in reducing softening point, but SSO and SO outperform PO in improving penetration. At 8% content, aged asphalt’s properties mostly recover to near-original levels, so 8% is the optimal dosage.
(2) The FTIR results show that the carbonyl index and sulfoxide index of aged asphalt decrease with the increase in the addition amount of the three bio-oils, indicating that the three bio-oils can improve the impact of aging on asphalt. In addition, at the optimal dosage, the regeneration efficiency of bio-oils on the chemical components of aged asphalt decreases in the order of SSO → SO → PO. The difference in regeneration effect between SO and PO is not significant, while SSO shows the best functional group repair ability.
(3) The results of the DSR test show that with increasing dosage of the three bio-oils, the complex shear modulus and rutting parameters of aged asphalt decrease while the phase angle increases. This restores the rheological properties of aged asphalt to a certain extent but impairs its high-temperature stability. The rutting parameter (G*/sin δ) of rejuvenated asphalt decreases systematically with higher bio-oil dosage; it drops significantly in the low-temperature range (40–60 °C) but the decreasing trend flattens in the high-temperature range (>60 °C). In conclusion, bio-oil dosage should be controlled, and the upper limit of their optimal dosage is recommended to be set at 8%.
(4) In this paper, molecular models of virgin asphalt, aged asphalt, bio-oil, and rejuvenated asphalt were established through molecular dynamics (MD). The density and solubility parameters of the virgin asphalt were obtained by MD. The simulation test results are consistent with the existing results, indicating the correctness of the established asphalt molecular model.
(5) The diffusion behavior analysis of three types of rejuvenated asphalts, LAA+SO, LAA+PO, and LAA+SSO, was studied through molecular dynamics simulation. By comparing the MSD values of the three rejuvenators, it can be found that the diffusion effects of soybean oil and palm oil are better than those of sunflower oil. Soybean oil and palm oil can effectively improve the performance of aged asphalt and enhance its diffusion ability in various components.
(6) Through the RDF simulation of the four components of asphalt, it was found that compared with sunflower oil, the g(r) peaks of each component in the rejuvenated asphalts with soybean oil and palm oil are higher, and the packing density of the asphalt molecular model is greater. This indicates that the orderliness of asphalt molecules is enhanced, and also demonstrates that there is good compatibility between soybean oil, palm oil, and asphalt.

Through the radar chart, the differences in the key performances of the three bio-oils can be clearly seen, which provides support for the subsequent development of a compound bio-oil rejuvenator with more balanced physical properties, rheological properties, and regeneration effects. Future research will focus on verifying the rationality of the compound vegetable oil rejuvenator and verifying the scientific nature of the design from an experimental perspective. We will more comprehensively evaluate the regeneration effect of the bio-oil rejuvenator on aged asphalt.

## Figures and Tables

**Table 1:** Physical properties of PEN 90 asphalt.

Property	Unit	Specification	Measured	Standard
Penetration at 25 °C	0.1 mm	80–100	96	ASTM-D5
Softening point	°C	≥44	44.3	ASTM-D36

**Table 2:** The number of bio-oil molecules and the mass ratio in the model.

Bio-Oils	Triglyceride (TG)	The Number in the Model	The Mass Ratio to the Long-Term Aged Asphalt
Soybean oil	LLL	2	11.9%
	LLLn	1	
	LLO	1	
	PLL	1	
Palm oil	LLL	2	11.1%
	LLO	2	
	PLL	1	
Sunflower oil	PPO	2	12.63%
	POO	2	
	POL	1	
	PPL	1	

**Table 3:** The parameters in fitting curves of different asphalt models.

Asphalt	Component	Linear Fitting	R^2^
Soybean oil-rejuvenated asphalt	Total	y = 1.47505 + 0.04052x	0.9737
	Aromatic	y = 1.07727 + 0.04751x	0.9937
	Asphaltene	y = 0.86399 + 0.03333x	0.8045
	Resin	y = 1.2091 + 0.03818x	0.9300
	Saturate	y = 3.24539 + 0.01438x	0.7915
	Soybean oil	y = 2.04913 + 0.06351x	0.9774
Palm oil-rejuvenated asphalt	Total	y = 1.15878 + 0.04668x	0.9724
	Aromatic	y = 1.86179 + 0.02913x	0.9672
	Asphaltene	y = 0.75469 + 0.03521x	0.8950
	Resin	y = 0.19314 + 0.06445x	0.9853
	Saturate	y = 2.33368 + 0.02157x	0.9020
	Palm oil	y = 1.35179 + 0.07199x	0.9222
Sunflower oil-rejuvenated asphalt	Total	y = 2.48185 + 0.02299x	0.8981
	Aromatic	y = 2.95593 + 0.0172x	0.77749
	Asphaltene	y = 2.19491 + 0.01067x	0.5615
	Resin	y = 1.6615 + 0.02843x	0.8413
	Saturate	y = 2.13703 + 0.03526x	0.9176
	Sunflower oil	y = 4.13658 + 0.02424x	0.7590
Virgin asphalt	Total	y = 1.57579 + 0.04352x	0.9441
	Aromatic	y = 1.71042 + 0.04537x	0.9293
	Asphaltene	y = 2.23989 + 0.02778x	0.9346
	Resin	y = 1.0842 + 0.04739x	0.9401
	Saturate	y = 1.74902 + 0.04794x	0.9396
Long-term aged asphalt	Total	y = 1.82592 + 0.02111x	0.9851
	Aromatic	y = 1.41404 + 0.02806x	0.9776
	Asphaltene	y = 1.49739 + 0.02429x	0.9620
	Resin	y = 2.23452 + 0.01047x	0.8760
	Saturate	y = 2.086 + 0.02965x	0.9659

**Table 4:** The original inputs for the five parameters.

Parameter	VA	LAA	LAA+SO	LAA+PO	LAA+SSO
diffusion coefficient	7.3	3.5	6.8	7.8	3.8
penetration	96	27.3	89	73.9	89.9
softening point	44.3	59.8	49.2	49.6	48.2
FTIR index	0.015	0.060	0.035	0.035	0.029
rutting parameter at 80 °C	236.31	1643.5	420.49	451.86	427.55
phase angle at 80 °C	89.16	83.04	85.29	85.44	85.47

## Data Availability

The original contributions presented in this study are included in the article. Further inquiries can be directed to the corresponding author.
